# Tuning band gap and enhancing optical functions of AGeF_3_ (A = K, Rb) under pressure for improved optoelectronic applications

**DOI:** 10.1038/s41598-022-12713-4

**Published:** 2022-05-23

**Authors:** Md. Safin Alam, Md Saiduzzaman, Arpon Biswas, Tanjun Ahmed, Aldina Sultana, Khandaker Monower Hossain

**Affiliations:** 1 Department of Materials Science and Engineering, Khulna University of Engineering & Technology (KUET), Khulna, 9203 Bangladesh; 2 Department of Materials Science and Engineering, University of Rajshahi, Rajshahi, 6205 Bangladesh

**Keywords:** Condensed-matter physics, Theory and computation

## Abstract

The current study diligently analyzes the physical characteristics of halide perovskites AGeF_3_ (A = K, Rb) under hydrostatic pressure using density functional theory. The goal of this research is to reduce the electronic band gap of AGeF_3_ (A = K, Rb) under pressure in order to improve the optical characteristics and assess the compounds’ suitability for optoelectronic applications. The structural parameters exhibit a high degree of precision, which correlates well with previously published work. In addition, the bond length and lattice parameters decrease significantly leading to a stronger interaction between atoms. The bonding between K(Rb)–F and Ge–F reveal ionic and covalent nature, respectively, and the bonds become stronger under pressure. The application of hydrostatic pressure demonstrates remarkable changes in the optical absorption and conductivity. The band gap becomes lower with the increment of pressure, resulting in better conductivity. The optical functions also predict that the studied materials might be used in a variety of optoelectronic devices operating in the visible and ultraviolet spectrum. Interestingly, the compounds become more suitable to be used in optoelectronic applications under pressure. Moreover, the external pressure has profound dominance on the mechanical behavior of the titled perovskites, which make them more ductile and anisotropic.

## Introduction

Cubic perovskites have obtained substantial preference from researchers and scientists over the last few years. The versatile applicability of these perovskites in multiple sectors, e.g., semiconductors, sensors, superconductivity, photovoltaic cells, optoelectronic devices, and LEDs (light-emitting devices)^1–3^ puts them in the center of attention. As a result, the researchers conducted both experimental and theoretical studies^4–7^ on the physical behavior of perovskite materials to create new possibilities for their applications in various optoelectronic fields. Interestingly, the improvement of perovskite solar cells (PSCs) has been accelerated, resulting in power conversion efficiency (PCE) of 22.1%^8^. Till now, the best PCE of 25.2% is recorded for Pb-based perovskite solar cells^9^ but have less life span caused by humidity, moisture, temperature, and UV light^10^. On the other hand, the lead-free tin halide perovskite incorporated with ethylammonium iodide exhibited the PCE of ~ 13%^11^. At initial stages, metallic Pb^2+^-based organic PSCs i.e., CH_3_NH_3_PbI_3_ (or MAPbX_3_) were developed^12–14^. But the toxicity of these organic compounds created major disadvantages. Pb is a recognized toxin, creating a number of obstacles^15^. In addition, organic MA^+^ cations cause serious environmental drawbacks, making the manufacturing process extremely risky and harmful^16^. In order to nullify the toxicity, non-toxic cations, like Ge^2+^ and Sn^2+^ have replaced Pb^2+^ cation, and/or K^+^, Rb^+^, and Cs^+^ cations have used to replace the organic counterpart^15,17–19^. Therefore, a new formation of ABX_3_ has appeared in which A, B, and C denote the monovalent cation, divalent cation, and halogen anion, respectively. Inorganic perovskites based on Ge have emerged as a possible alternative of Pb, because they possess superior optical absorption and conductivity as compared to Pb-based perovskites^20^. At ambient temperature, Ge-based perovskites do not exhibit any phase transformation^21,22^. Besides, K and Rb have shown promising potential for photo-voltaic applications^23^. Jain et al.^24^ have utilized the first-principle calculations on RbSn(Cl,Br)_3_ perovskites to evaluate prominent band gap suitable for photovoltaics. The monovalent cation K^+^-based perovskites have potential to be utilized in solar cells because of high absorption and configurable band gap^20,22,25^. In recent studies, the inorganic halide perovskites have been recognized as a reliable material for solar cell applications^25,26^. For the purpose of enhancing physical properties of halide perovskites, the application of hydrostatic pressure has demonstrated tremendous results^27–32^. Usually, hydrostatic pressure modifies the lattice parameters^33^, displacement of cation and anion^34,35^, rotation of octahedral cages^36^, phase transitions^37,38^, etc. In the case of metal halides, structural properties, like lattice constants and unit cell volume decrease with increasing pressure^29,32^. Identical behavior can be detected in halide perovskites as well. In recent works, inorganic halide perovskites, such as KCaCl_3_^39^, CsGeI_3_^30^, RbYbF_3_^40^, and CsGeI_3_^41^ have shown reduction in band gap under hydrostatic pressure, resulting an improvement of conductivity. In addition, the application of pressure can remarkably develop the optical parameters of halide perovskites, enhancing the functionality in optoelectronic fields. Therefore, the motive of present work is to evaluate and examine the changes of various physical features of halide perovskites AGeF_3_ (A = K, Rb) after applying hydrostatic pressure. More specifically, this study has analyzed the structural, electronic, optical, and mechanical properties of AGeF_3_ (A = K, Rb) using first-principle calculations to observe whether the application of hydrostatic pressure has made them more appealing to optoelectronic fields or not.

## Results and discussion

### Structural properties

**Figure 1:**
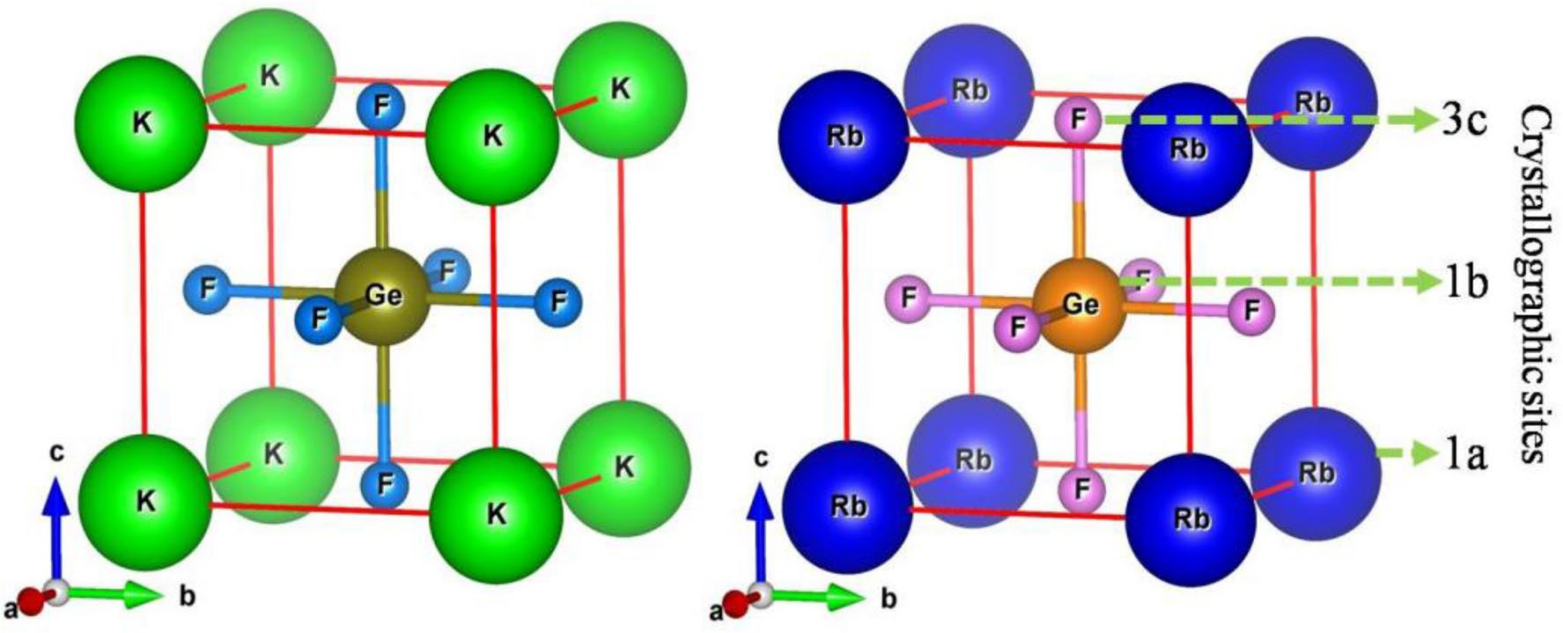
The optimized crystal structure of halide perovskites KGeF_3_ and RbGeF_3_.

The geometry optimization states that the selected compounds AGeF_3_ (A = K, Rb) under study crystallized in cubic cell that have the space group *Pm-*3*m* (#221). In the unit cell, the A (= K, Rb), Ge, and F atoms are located at the corner, body center, and face center, respectively, with the Wyckoff positions 1a (0, 0, 0), 1b (0.5, 0.5, 0.5), and 3c (0, 0.5, 0.5), respectively. The optimized crystal structure of AGeF_3_ (A = K, Rb) with crystallographic sites is illustrated in Fig. 1. The evaluated lattice constant of KGeF_3_ at ambient pressure is 4.451 Å (Table 1), which is relatively closer to the reference study (4.46 Å)^20^. The deviation value of 0.2% presents the high accuracy of this study. For RbGeF_3_, the lattice constant is 4.490 Å (Table 1), showing no deviation from the previous work (4.49 Å)^20^. The hydrostatic pressure ranging from 0 to 30 GPa is applied on both compounds to calculate the structural parameters as given in Table 1. The application of pressure demonstrates a significant effect on the structural parameters. The changes of relative lattice constants and unit cell volume with respect to the hydrostatic pressure are illustrated in Fig. S1a,b, respectively. The plotted graphs reveal the reduction of both lattice constant and unit cell volume under linear ascending of applied pressure. This decreasing tendency of lattice parameters under hydrostatic pressure indicates the reduction of bond length (Table 2) within the compounds. In order to justify the phase stability of AGeF_3_ (*A* = K, Rb) under pressure, the formation energy (Δ*E*_f_) is calculated using the following equation and recorded in Table 1.

1 $${\Delta E}_{\mathrm{f}}\left({\mathrm{AGeF}}_{3}\right)=\frac{\left[{E}_{tot.}\left({\mathrm{AGeF}}_{3}\right) - {E}_{s}\left(\mathrm{A}\right) - {E}_{s}\left(\mathrm{Ge}\right) - 3{E}_{s}\left(\mathrm{F}\right)\right]}{N}.$$

Here, *E*_s_(A), *E*_s_(Ge), and *E*_s_(F) are the energy of A (= K, Rb), Ge, and F atoms, respectively, whereas, *E*_tot_(AGeF_3_) represents the unit cell total energy of AGeF_3_, and *N* is the number of atoms in the unit cell. The negative values of Δ*E*_f_ at all applied pressures reveal the thermodynamic stability of titled halide systems^42^.

**Table 1:** Calculated lattice constant (*a*), unit cell volume (*V*), and formation energy (Δ*E*_f_) of AGeF_3_ (A = K, Rb) at various applied pressures.

Compound	Calculated data	Pressure (GPa)			
		0	10	20	30
KGeF_3_	*a* (Å)	4.451 [This work]	4.243	4.117	4.023
		4.46^31^			
	*V* (Å^3^)	88.18	76.37	69.78	65.11
	Δ*E*_f_ (eV/atom)	− 4.63	− 4.57	− 4.44	− 4.30
RbGeF_3_	*a* (Å)	4.490 [This work]	4.281	4.154	4.061
		4.49^31^			
	*V* (Å^3^)	90.52	78.46	71.68	66.97
	Δ*E*_f_ (eV/atom)	− 4.62	− 4.55	− 4.43	− 4.28

**Table 2:** Estimated bond lengths in AGeF_3_ (A = K, Rb) at various applied pressures.

Pressure (GPa)	Bond length (Å)			
	KGeF_3_		RbGeF_3_	
	Ge–F	K–F	Ge–F	Rb–F
0	3.14714	2.22537	3.17491	2.24500
10	3.00043	2.12162	3.02725	2.14059
20	2.91083	2.05827	2.93745	2.07709
30	2.84527	2.01191	2.87188	2.03072

### Electronic properties

**Figure 2:**
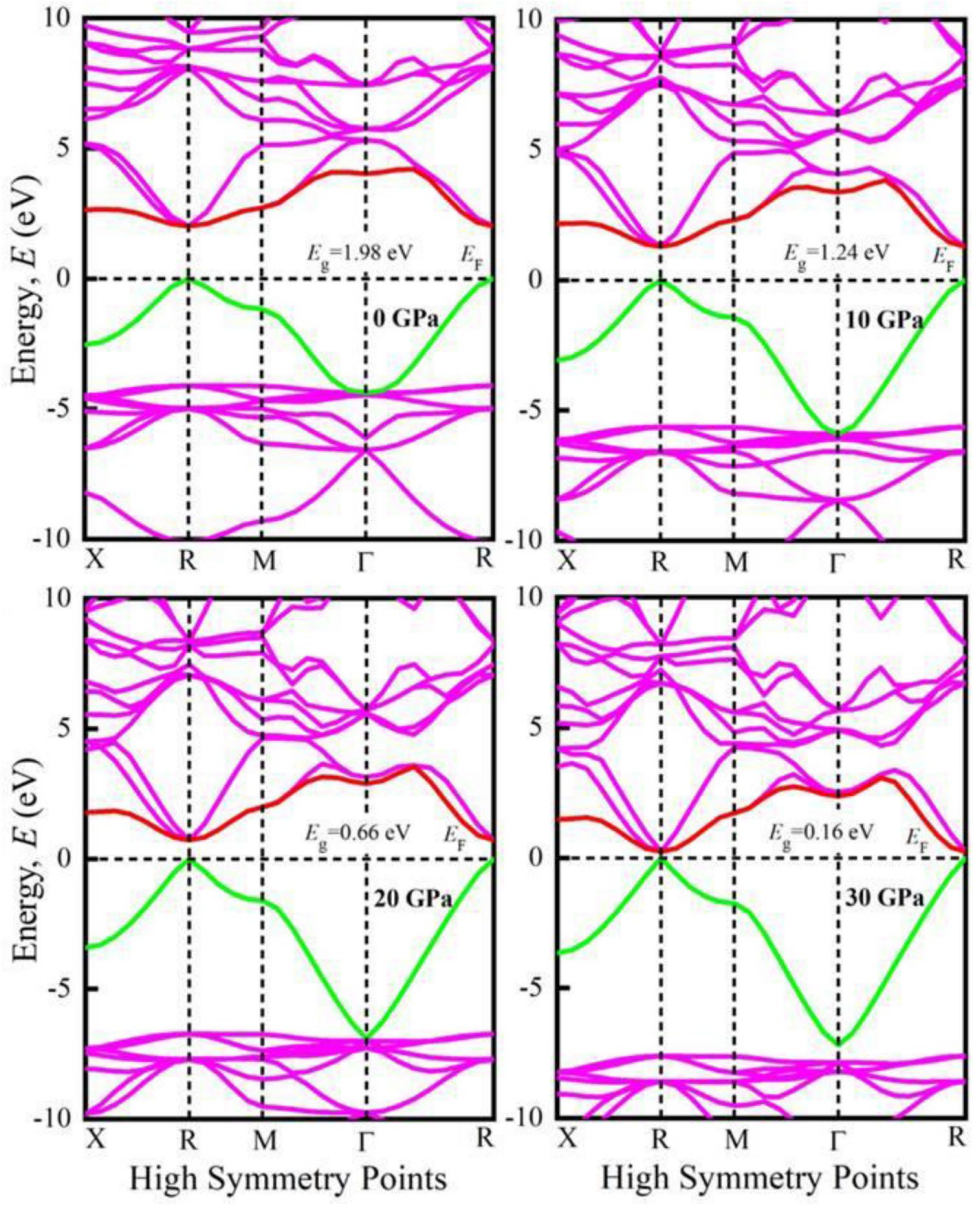
Band structures of KGeF_3_ under applied pressure.

**Figure 3:**
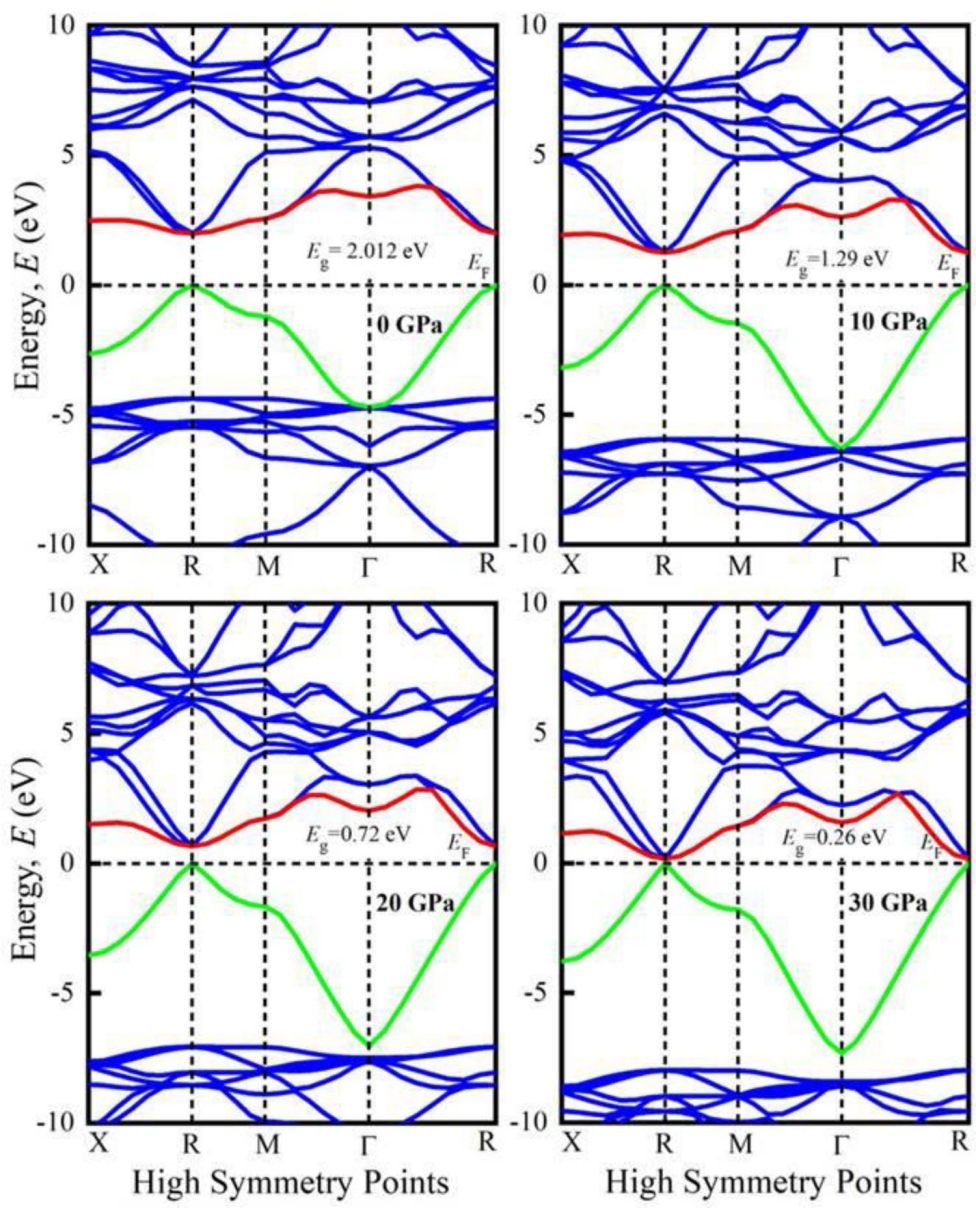
Band structures of RbGeF_3_ under applied pressure.

The assessment a material’s electronic nature requires the understanding of its band structure and density of states. The band structures of KGeF_3_ and RbGeF_3_ at different applied pressures are shown in Figs. 2 and 3, respectively. The horizontal dotted line at 0 eV denotes the Fermi level (*E*_F_). At 0 GPa, the valence band maximum (VBM) and conduction band minimum (CBM) indicated by green and red lines, respectively, of both compounds are noticed at R point of the Brillouin zone. Therefore, a direct band gap (*E*_g_) of 1.98 eV is found for KGeF_3_, while it is 2.012 eV for RbGeF_3_. The *E*_g_ values found for AGeF_3_ (A = K, Rb) are quite consistent with the theoretical values obtained using the GGA-PBE approximation^19^. With increasing pressure, the CBM of both compounds begin to move towards the *E*_F_, resulting in a reduction of *E*_g_. At 30 GPa, the *E*_g_ of KGeF_3_ falls to 0.16 eV, while it is 0.26 eV for RbGeF_3_. The reduction of *E*_g_ under pressure for both compounds is graphically presented in Fig. S2. There exists an inverse relationship between band gap and external pressure^43^, which can increase the potential between electron and ion responsible for reducing lattice parameters (Table 1). The band gap at the Brillouin zone symmetry point shrinks when the lattice parameter is reduced. The reduction of *E*_g_ allows easy transport of electrons from valence band to conduction band. As a result, the optical absorption and conductivity may become higher beneficial for optoelectronic applications.

**Figure 4:**
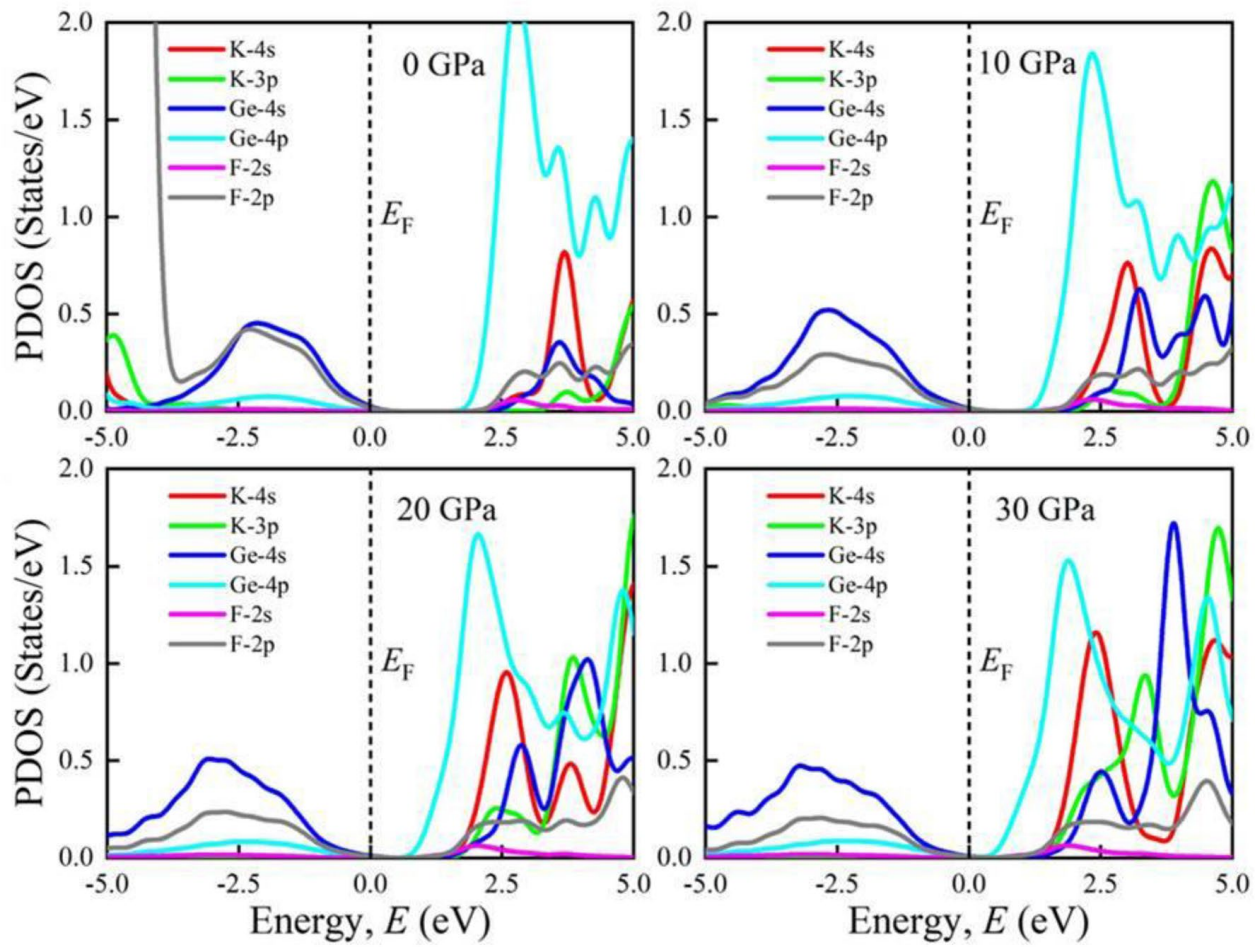
Partial density of states of KGeF_3_ under applied pressure.

**Figure 5:**
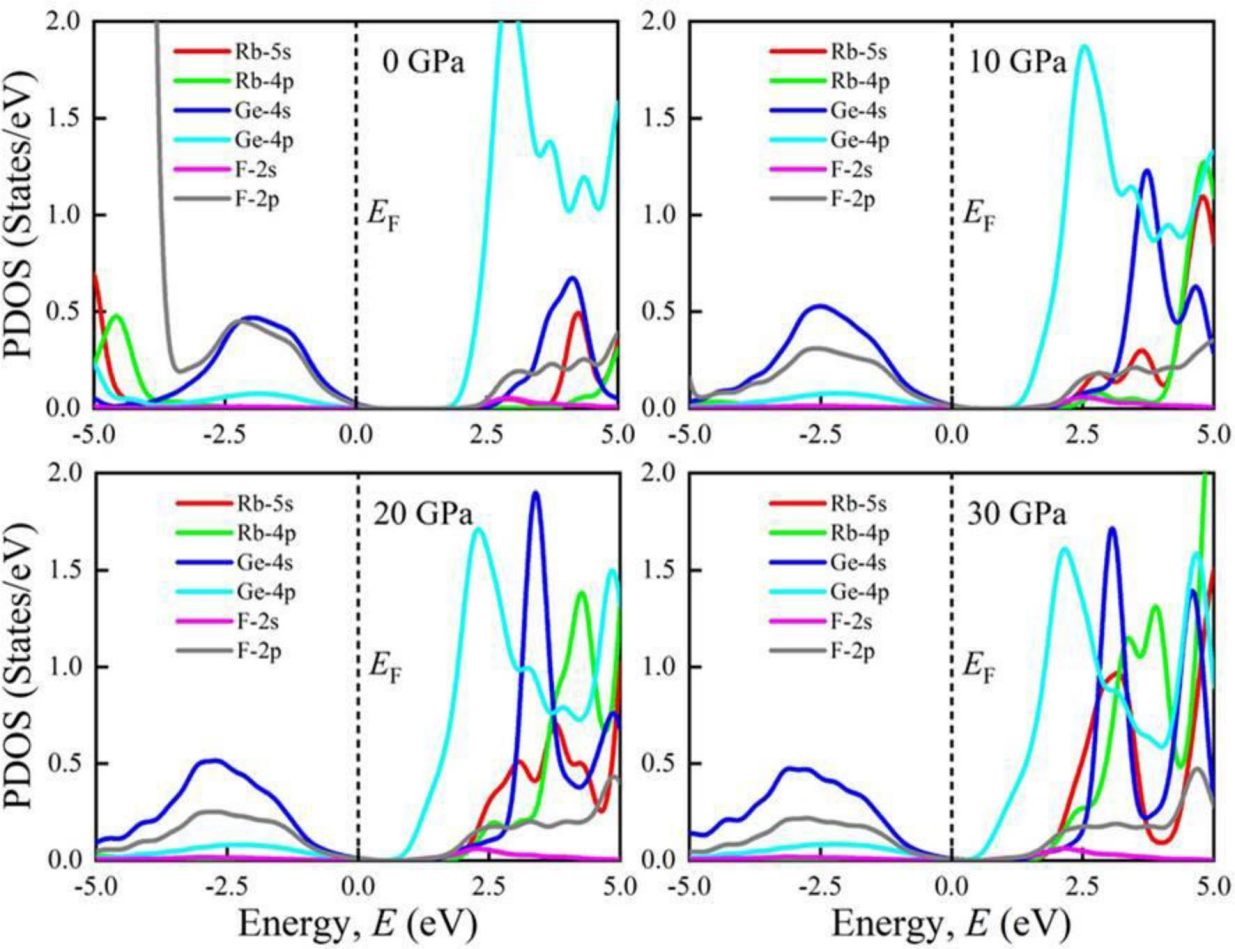
Partial density of states of RbGeF_3_ under applied pressure.

Furthermore, the total density of states (TDOS) of AGeF_3_ (A = K, Rb) are computed and illustrated in Fig. S3a,b to explicate the band structures. The vertical dashed line at 0 eV denotes the *E*_F_. There observe no TDOS value at *E*_F_ for both compounds under all applied pressures, which also reflects the semiconducting nature of them. There is a significant pressure influence on TDOS in the conduction band, where all the sharp peaks gradually move towards the *E*_F_ as pressure increases. This peak shifting is responsible for the band gap shrinking under pressure, which is also appeared in the band structures at R point. However, the partial density of states (PDOS) is crucial to obtain the atomic contribution of a material for making its band structure. It is evident from Figs. 4 and 5 that the valence band of both compounds near the *E*_F_ mostly originate from Ge-4s and F-2p orbitals with small amount of Ge-4p orbital. On the other hand, the conduction band results from K-4s (Rb-5s), K-4p (Rb-4p), Ge-4s, Ge-4p, and F-2p states. It is apparent that the Ge-4p orbital is mainly responsible for narrowing the *E*_g_ in both compounds. The hybridization between Ge-4p and F-2p is promoted by raising external pressure, which raises the conduction bands towards the *E*_F_ and reduces the band gap. Furthermore, the shortening of Ge–F bond length in response to pressure (Table 2) could improve the hybridization between Ge-4p and F-2p orbitals in the conduction band, which lowers the CBM at R point of the Brillouin zone (Figs. 2, 3). Hence, the band gap of KGeF_3_ (RbGeF_3_) reduces from 1.98 (2.012 eV) to 0.16 eV (0.26 eV).

**Figure 6:**
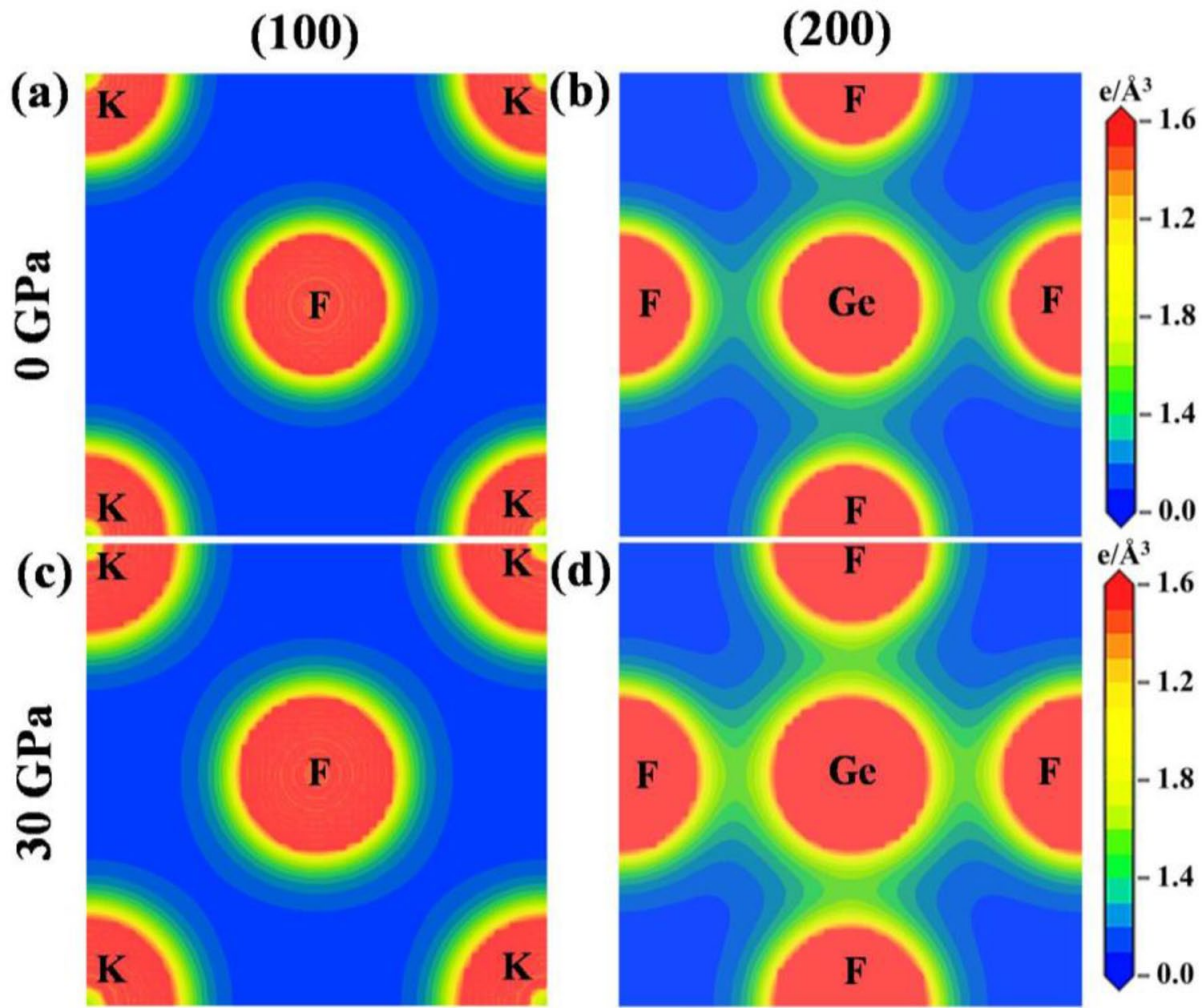
Charge density plots of KGeF_3_ at 0 GPa and 30 GPa pressure.

**Figure 7:**
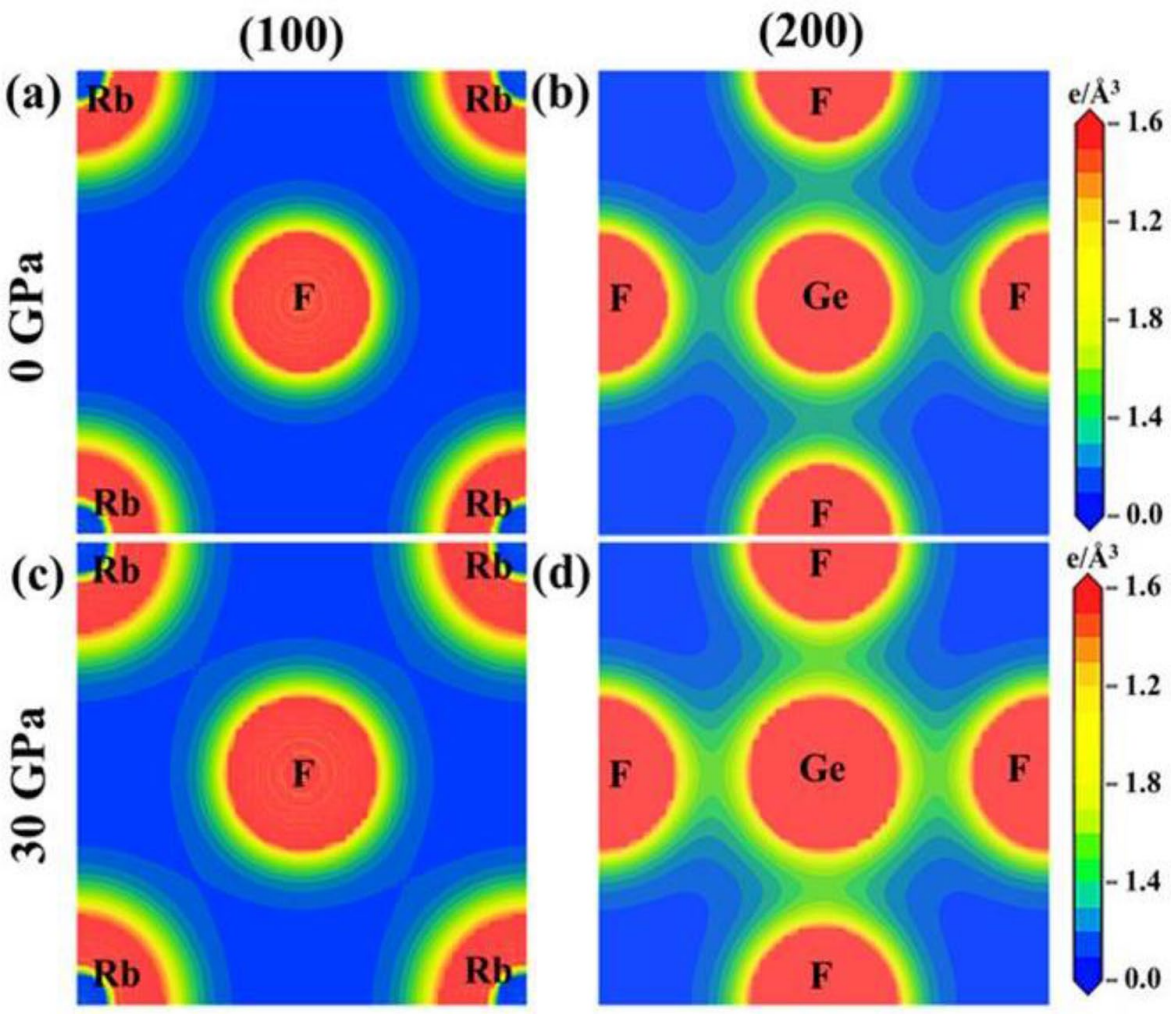
Charge density plots of RbGeF_3_ at 0 GPa and 30 GPa pressure.

Charge density mapping helps to visualize the charge distribution around atoms and bonding nature of the compounds. Figures 6 and 7 illustrate the charge density mapping of AGeF_3_ (A = K, Rb) along the crystallographic planes (100) and (200). The right side scales indicate the electron density, in which low and high intensity are imparted by blue and red colors, respectively. At ambient pressure, K(Rb) and F atoms exhibit spherical charge contours along the (100) plane, manifesting the existence of ionic bonding between them (Figs. 6a, 7a). In addition, a covalent bonding nature of Ge–F is predicted, as the elliptical shape of charge distribution is observed around Ge and F atoms along the (200) plane (Figs. 6b, 7b). Charge density is also estimated at an applied pressure of 30 GPa to understand the effect of pressure on charge distribution. There is no noticeable difference in the spherical charge contours around K(Rb) and F atoms along the (100) plane (Figs. 6c, 7c). However, the contours around Ge and F atoms become more elliptical along the (200) plane, intensifying the covalent bonding of Ge–F (Figs. 6d, 7d). The bond length of Ge–F is longer than that of K(Rb)–F (Table 1), indicating strong bonding between K(Rb) and F atoms than that of bonding between Ge and F atoms. Therefore, the weaker covalent bond of Ge–F and stronger ionic bond of K(Rb)–F are exhibited in the crystal structure of AGeF_3_ (A = K, Rb), which validates the estimated results predicted by charge density maps. The bond length decreases monotonically as pressure increases (Fig. S4a,b) and hence, the ionic/covalent bonds become stronger.

### Optical properties

Metal halides without lead (non-toxic) have served as a source of interest due to their outstanding optical characteristics. They demonstrate much appreciated performance in optoelectronic devices and photovoltaic cells. This section deeply analyzes and discusses a few optical characteristics, namely absorption, conductivity, reflectivity, reflective index, and dielectric function. The findings are presented using an electronic polarization vector of [100] at 0 GPa and 30 GPa pressures.

It is necessary to determine dielectric function to obtain rest of the optical functions^44^. It can be represented as *ε*(ω) = *ε*_1_(ω) + *iε*_2_(ω); where *ε*_1_(ω) and *ε*_2_(ω) denote the real and imaginary parts of dielectric function, respectively^45^. According to Kramers–Kronig relation^46^, *ε*_1_(ω) is written as,

$$\varepsilon_{1 } \left( \omega \right) = 1 + \frac{2}{\pi }P\mathop \smallint \limits_{0}^{\infty } \frac{{\omega^{{^{\prime}\varepsilon_{2} }} \left( {\omega^{\prime}} \right)}}{{\omega^{^{\prime}2} - \omega^{2} }}d\omega^{\prime}.$$

On the other hand, *ε*_2_(ω) may also calculate using the momentum tensors between the occupied and unoccupied wave functions^47,48^.

$${\varepsilon }_{2}\left(\omega \right)= \frac{{2e}^{2}\pi }{\Omega {\varepsilon }_{0}}\sum \limits_{K,V,C}{\left|\langle {\psi }_{k}^{c}\left|\widehat{U}\right. \cdot \overrightarrow{r}\left|{\psi }_{k}^{V}\right.\rangle \right|}^{2}\delta ({E}_{K}^{C}- {E}_{K}^{V}-E).$$

Here, *ω* signifies the light frequency. ${\psi }_{k}^{c}$ and ${\psi }_{k}^{V}$ denote the conduction and valance band wave function at *k*, respectively, *e* is the electronic charge, Ω represents the unit cell volume, and *U* indicates the unit vector along the polarization of the incident electric field. The delta function ensures energy and momentum conservation during a transition between occupied and unoccupied electronic states through the emission or absorption of photon energy, *E*. ${E}_{K}^{C}$ and ${E}_{K}^{V}$ denote the energy of electrons at a certain *k-*vector in the conduction and valence bands, respectively. The rest of the optical parameters are calculated using the expressions given elsewhere^49^.

**Figure 8:**
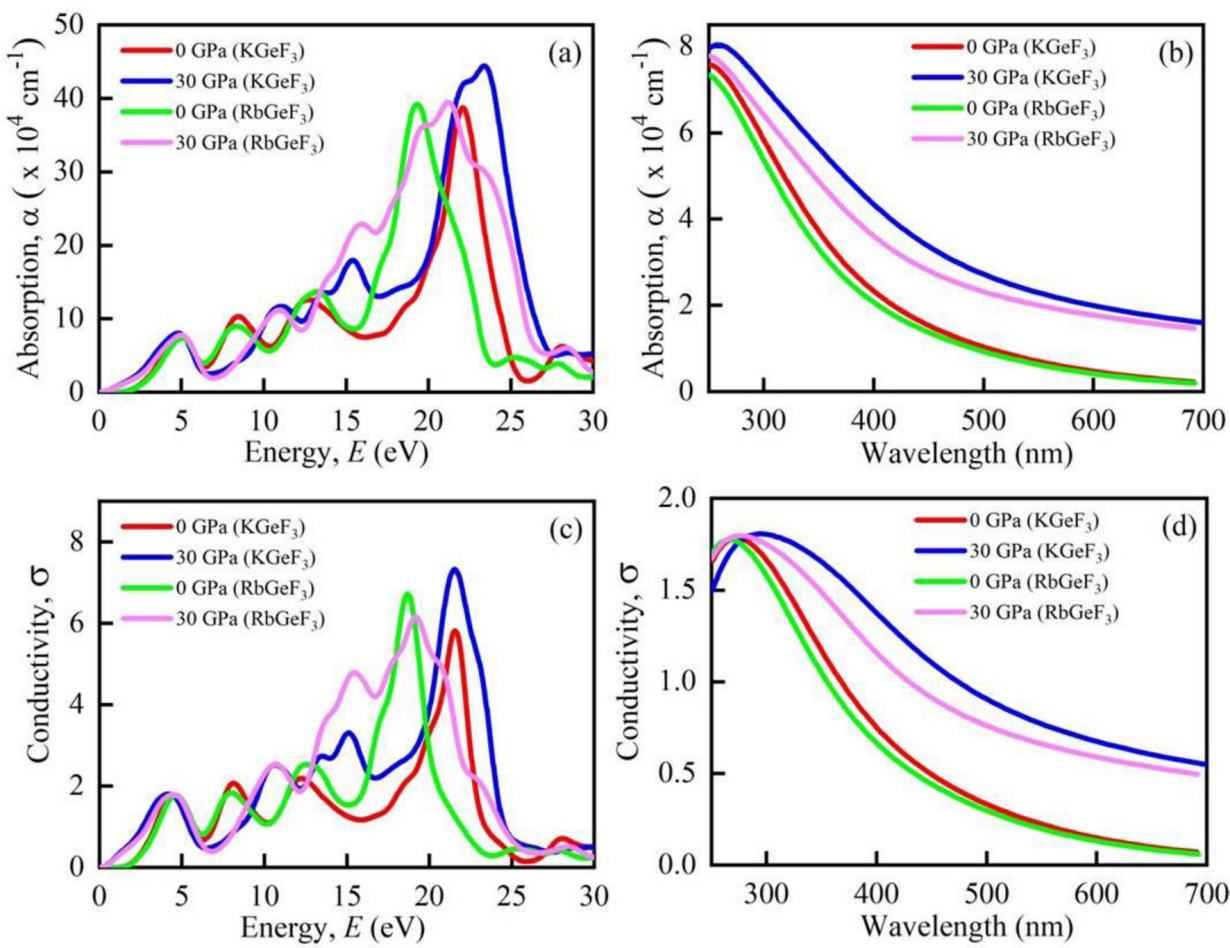
Calculated pressure induced optical **(a)** absorption vs. energy, **(b)** absorption vs. wavelength, **(c)** conductivity vs. energy, and **(d)** conductivity vs. wavelength of AGeF_3_ (A = K, Rb).

The optical absorption coefficient (*α*) denotes the amount of energy absorbed by a substance per unit length. The efficiency of a material's optimal solar energy conversion can simply be described by it. From Fig. 8a, the absorption does not begin at 0 eV, since both KGeF_3_ and RbGeF_3_ contain a band gap at ambient pressure. In the ultraviolet region, KGeF_3_ and RbGeF_3_ show three sharp peaks in the range of ~ 8–22 eV and ~ 8–18 eV, respectively. So, both compounds work as good absorbers in the ultraviolet region at 0 GPa. When 30 GPa pressure is applied both compounds also show peaks within the ultraviolet region similar that exhibit at 0 GPa. In this case, both compounds show an additional peak at ~ 15 eV. Interestingly, the absorption spectra almost start from 0 eV (but not 0 eV due to having very small band gap) at 30 GPa (inset of Fig. 8a). The absorption in the visible light region is explicitly shown in Fig. 8b. The absorption of both compounds in the visible region is explicitly higher at 30 GPa than that observed at 0 GPa. As a result, the studied perovskites are expected to use visible light energy for photovoltaic conversion at a pressure of 30 GPa, potentially increasing the efficiency of solar cells.

**Figure 9:**
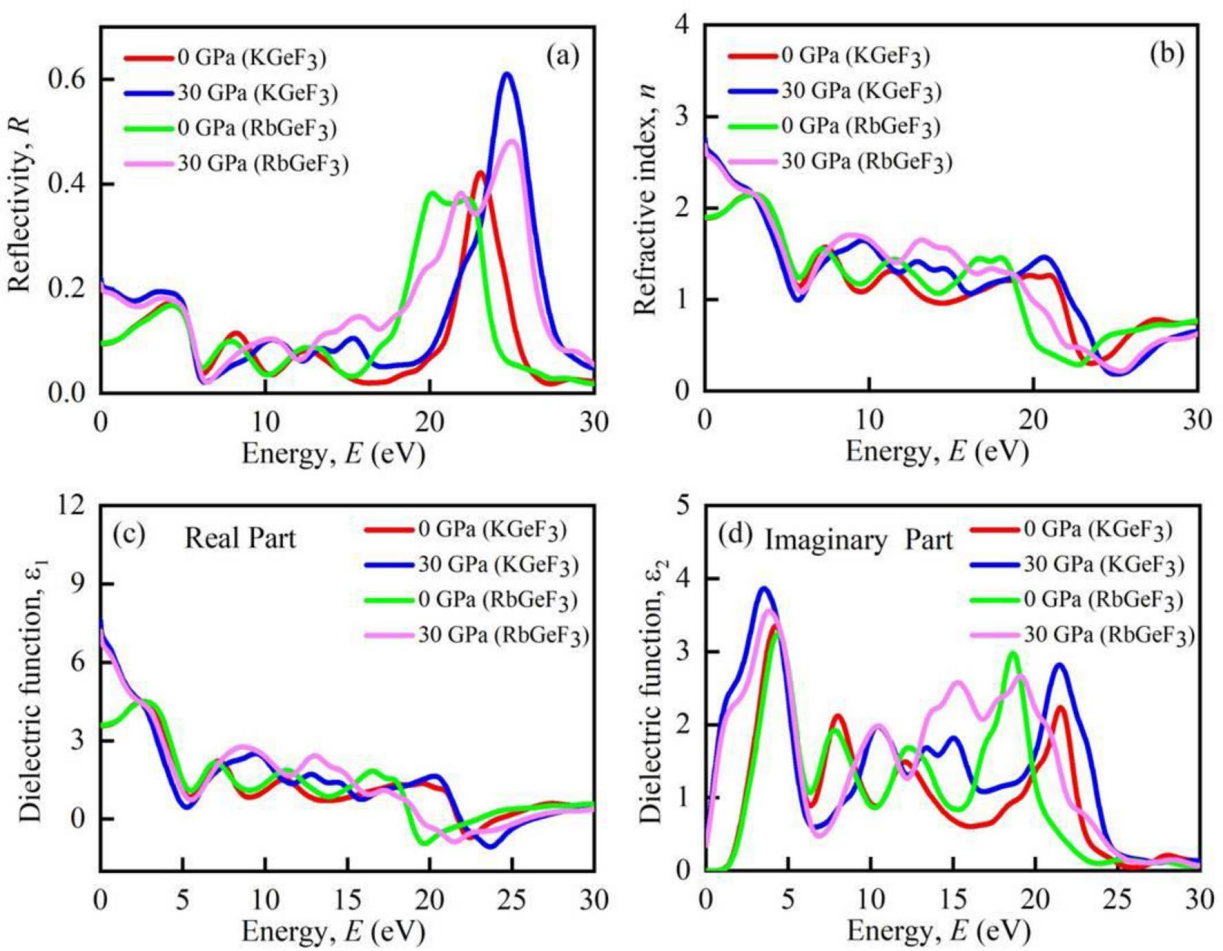
Calculated pressure induced spectra of optical **(a)** reflectivity, **(b)** refractive index, **(c)** real part of dielectric function, and **(d)** imaginary part of dielectric function of AGeF_3_ (A = K, Rb).

Photoconductivity is another term of optical conductivity (*σ*). It refers to the conductivity of photons in a substance^50^. Figure 8c illustrates the *σ* of AGeF_3_ (A = K, Rb) at 0 GPa and 30 GPa pressures. At 0 GPa, the perovskites exhibit *σ* in the visible area. The *σ* achieves its maximum value at negative *ε*_1_(ω) (Fig. 9c). Therefore, KGeF_3_ and RbGeF_3_ show maximum *σ* in the energy region ~ 22–24 eV and ~ 18–22 eV, respectively. At 30 GPa, both compounds show increased *σ* in the visible region (Fig. 8d). Just like at 0 GPa, the highest *σ* peak of KGeF_3_ is in the energy region ~ 22–24 eV. But the highest *σ* peak slightly shifts to the energy range ~ 19–21 eV for RbGeF_3_. However, the *σ* of both compounds significantly increases in the visible region under pressure as can be seen in Fig. 8d because of increased absorption.

The reflectivity (*R*) is a critical optical feature for material’s photovoltaic applications. When exposed to photon with very low energy, KGeF_3_ and RbGeF_3_ reflect around 10% of the incident light (Fig. 9a). It rises for both substances when they transit from the infrared to the visible range. After transiting to the ultraviolet region, KGeF_3_ and RbGeF_3_ exhibit their highest *R* peak at ~ 23 eV and ~ 20 eV, where the dielectric function’s real part is negative. Under 30 GPa pressure, the *R* of both compounds spikes to 20% at zero energy. The application of pressure increases the *R* of both materials throughout almost whole energy regions. However, the relatively lower *R* (with or without the application of pressure) at low energy region indicates the potentiality of both compounds in solar cell applications. Additionally, both compounds should be applied as coating material to minimize solar heating because of their higher *R* at high energy region^51^.

The refractive index (*n*) is used to determine the amount of light bent or refracted as it enters into a substance. Furthermore, the phase velocity of an electromagnetic wave in a medium can also be calculated by *n*. According to Fig. 9b, both compound’s *n* is prominent at low energy and showing a fluctuating nature in the high energy region. When 30 GPa pressure is applied, the *n* of both compounds significantly enhances at 0 eV. This implies that AGeF_3_ (A = K, Rb) should be preferable for optical devices, like photonic crystals and waveguides^52^.

The dielectric function characterizes the interaction of a material with incoming electromagnetic radiation. As a result, it is critical to have knowledge about dielectric function for optoelectronic device applications. The real (*ε*_1_) and imaginary (*ε*_2_) parts of dielectric function of AGeF_3_ (A = K, Rb) are shown in Fig. 9c,d, respectively. The static dielectric function, *ε*_1_ (0) is an important quantity, which measures the efficiency of an optoelectronic device^15^. A material with a greater *ε*_1_ (0) has a lower rate of charge recombination, which results in improved performance of optoelectronic devices^52^. At ambient pressure, both compounds show identical *ε*_1_ low energy, which enhances in the infrared–visible region and declines upon entering into the ultraviolet region. In addition, both KGeF_3_ and RbGeF_3_ show negative *ε*_1_ at energy ranging from ~ 22 to 24 eV to ~ 18 to 22 eV, respectively. This implies that the compounds show high reflectivity at that energy region, which is evident in Fig. 9a. When 30 GPa pressure is applied, the *ε*_1_ (0) is remarkably increased owing to the lower recombination of charges, which makes the compounds even more suitable for optoelectronic devices applications. At 0 GPa, the *ε*_2_ is higher in the visible and early ultraviolet region, conveying high absorption at that regions^53^. But the spectrum of the *ε*_2_ shifts to the low energy region at high pressure. Specifically, the larger *ε*_1_ and *ε*_2_ at low energy together with smaller *ε*_1_ and *ε*_2_ at high energy areas evident the feasibility of both compounds in microelectronics and integrated circuits^39^, and the superiority is greatly enhanced under pressure.

### Mechanical properties

The elastic constants (*C*_ij_) are usually used to determine the structural stability and mechanical characteristics of a material. The elastic nature describes how a material deforms under strain before recovering and returning to its original shape once the load is removed. It is important to reveal information about the binding properties between adjoining atomic planes, the anisotropic nature, and structural stability^54^. A cubic compound has three independent elastic constants: *C*_11_, *C*_12_, and *C*_44_. Table 3 lists the computed *C*_11_, *C*_12_, *C*_44_, and Cauchy pressure (*C*_12_ − *C*_44_) for these two compounds under pressure. The elastic constants at ambient pressure are comparable with reported study^20^ but rise linearly as pressure increases (Fig. S5a). Since the well-known Born stability requirements (*C*_44_ > 0, *C*_11_ − *C*_12_ > 0, and *C*_11_ + 2*C*_12_ > 0)^55^ are nicely matched by all the calculated elastic constants, both the studied compounds are mechanically stable even under applied pressure. In addition, *C*_12_ − *C*_44_ can identify the brittleness and ductility of materials. If *C*_12_ − *C*_44_ possesses a positive (negative) value, the material should be ductile (brittle)^56^. Therefore, the titled compounds are expected to be ductile because of having positive values of *C*_12_ − *C*_44_ (Table 3). However, KGeF_3_ is slightly more ductility than that of RbGeF_3_ (Table 3).

**Table 3:** Calculated elastic constants *C*_ij_ (GPa) and Cauchy pressure *C*_12_ − *C*_44_ (GPa) of AGeF_3_ (A = K, Rb) at various applied pressures.

Pressure (GPa)	Compound	*C*_11_	*C*_12_	*C*_44_	*C*_12_ − *C*_44_
0^31^	KGeF_3_	80.67	35.57	5.96	29.61
	RbGeF_3_	82.61	34.28	12.53	21.75
0	KGeF_3_	93.66	30.17	13.07	17.10
	RbGeF_3_	91.35	33.25	16.53	16.72
10	KGeF_3_	165.95	52.47	10.22	42.25
	RbGeF_3_	163.40	57.57	16.45	41.12
20	KGeF_3_	233.93	77.25	6.66	70.59
	RbGeF_3_	227.88	81.06	15.51	65.55
30	KGeF_3_	293.88	98.42	2.51	95.91
	RbGeF_3_	292.15	107.79	13.99	93.80

Various essential mechanical characteristics, such as bulk modulus (*B*), shear modulus (*G*), Young’s modulus (*E*), Poisson’s ratio (*v*), Pugh’s ratio (*B/G*), and Zener anisotropy index (*A*) of AGeF_3_ (A = K, Rb) are determined using the estimated *C*_ij_ and presented in Table 4 with available reported data^20^. The *B* and *G* are determined using the Voigt–Reuss scheme. The Voigt and Reuss coefficients describe the upper and lower bounds of the effective modulus, respectively. For cubic lattices, the Voigt bulk modulus (*B*_V_) and Voigt shear modulus (*G*_V_) as well as the Reuss bulk modulus (*B*_R_) and Reuss shear modulus (*G*_R_) are described by the well-known expressions^57,58^. According to Hill’s theory^59^, the *B* and *G* are the arithmetic mean of Voigt and Reuss expressions. Furthermore, the *E* and *v* are provided by the equations reported elsewhere^59^. The *B* and *G* stand for fracture resistant and plastic deformation, respectively. Because of having greater *B* and *G*, RbGeF_3_ is more fracture and plastic deformation resistant than KGeF_3_ (Fig. S5b). *E* is a measure of material’s stiffness and has proportional relationship. As a result, RbGeF_3_ will be stiffer than KGeF_3_. However, the application of pressure induces more resistance to fracture and plastic deformation as well as makes them stiffer than that exhibited by the compounds without pressure. The variation of elastic moduli under applied pressure is graphically represented in Fig. S5b.

**Table 4:** The calculated bulk modulus *B* (GPa), shear modulus *G* (GPa), Young’s modulus *E* (GPa), Poisson’s ratio *v*, Pugh’s ratio *B*/*G*, and Zener anisotropy index *A* of AGeF_3_ (A = K, Rb) at various applied pressures.

Pressure (GPa)	Compound	*B*	*G*	*E*	*B*/*G*	*v*	*A*
0^31^	KGeF_3_	50.61	10.52	29.53	4.81	0.40	0.26
	RbGeF_3_	50.39	16.35	44.27	3.08	0.35	0.52
0	KGeF_3_	51.34	18.81	50.30	2.73	0.366	0.412
	RbGeF_3_	52.62	20.76	55.03	2.49	0.326	0.569
10	KGeF_3_	90.30	22.01	61.08	4.10	0.387	0.180
	RbGeF_3_	92.97	26.85	73.47	3.46	0.368	0.311
20	KGeF_3_	129.4	22.92	64.93	5.65	0.416	0.085
	RbGeF_3_	130.00	30.67	85.29	4.24	0.391	0.211
30	KGeF_3_	163.57	22.35	64.14	7.32	0.434	0.026
	RbGeF_3_	169.24	33.22	93.55	5.09	0.407	0.152

The critical value of *v* to distinguish a materials’ ductile or brittle nature is 0.26^60^. A material is said to be ductile if *v* is larger than 0.26. Thus, both KGeF_3_ and RbGeF_3_ are concluded as ductile materials (Table 4). Another essential feature is *B/G*, which has a crucial value of 1.75 to divide solid materials into ductile or brittle^61^. The calculated values of *B/G* also reveal the ductile behavior of both compounds (Table 4). However, the ductility of KGeF_3_ is slightly higher than that of RbGeF_3_. The ductility of the studied compounds at 0 GPa has previously been predicted^20^, which is consistent with this study. It seen from Figs. S6a,b that both *v* and *B/G*, respectively, are increased with increasing pressure, which exhibit more ductile nature of the studied compounds under pressure. Interestingly, the outcome of *v* and *B/G* completely resemblances the data of *C*_12_ − *C*_44_ (Tables 3, 4).

**Figure 10:**
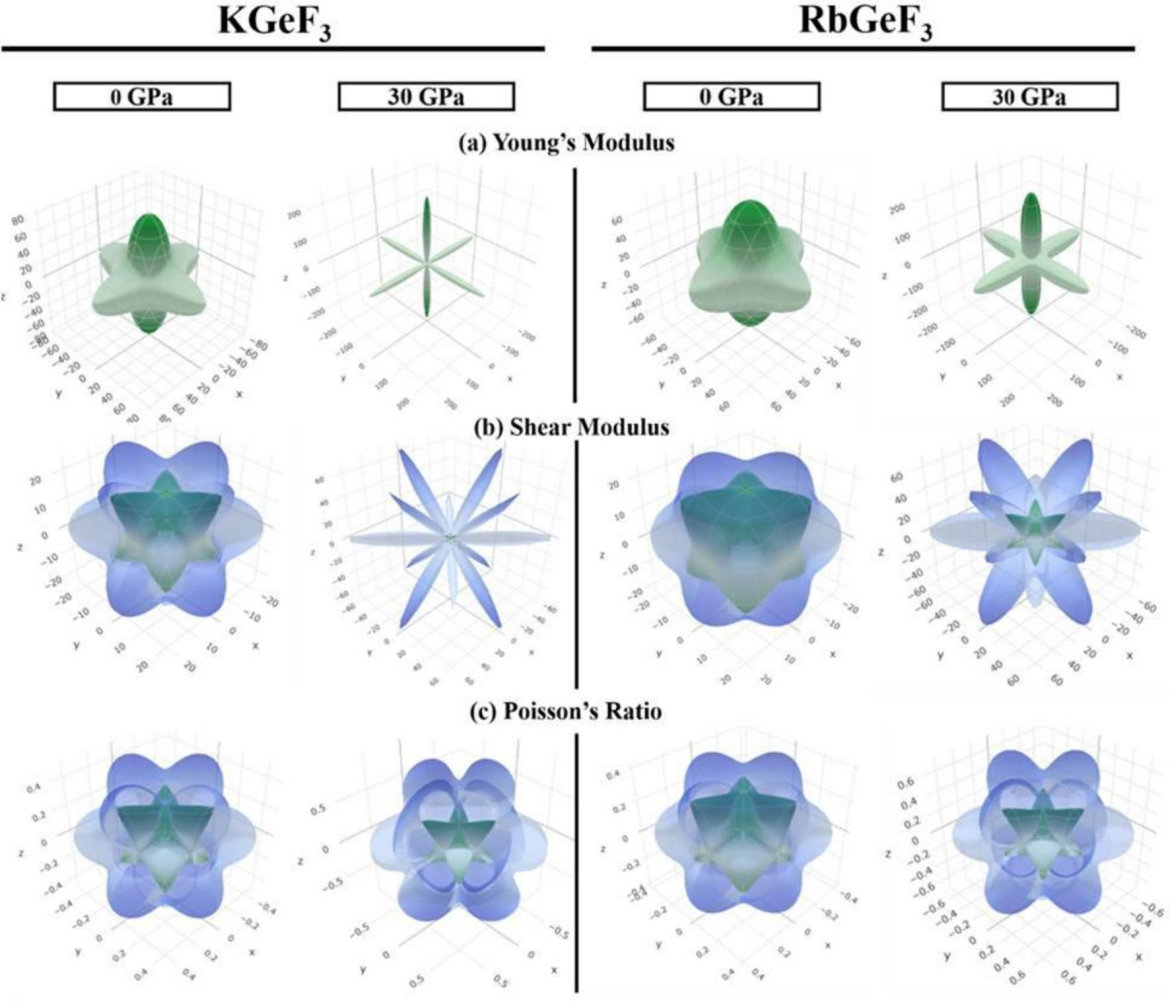
Anisotropic 3D representation of **(a)** Young’s modulus, **(b)** shear modulus, and **(c)** Poisson’s ratio of AGeF_3_ (A = K, Rb) at 0 and 30 GPa pressure.

In applied engineering, the ability to observe the influence of elastic anisotropy on these features is critical^62^. The properties of a system may be directionally dependent and anisotropic index is used to calculate it. For example, the shear anisotropic factor is utilized to determine the degree of anisotropy in the bonding strength of atoms along different crystallographic planes. Three shear anisotropic factors *A*_1_, *A*_2_, and *A*_3_ have been found along the (100), (010), and (001) planes, respectively^63^. For cubic systems, these are similar to the Zener anisotropy factor (*A*) and can be determined by the empirical formula^64^. An isotropic material must have the unit value of *A* and the departure of unity denotes anisotropy^65^. Both the compounds exhibit anisotropic nature, which are enhanced under pressure (Table 4). However, KGeF_3_ show more anisotropy as compared to KGeF_3_. Figure 10a–c show the direction dependence of *E*, *G*, and *v*, respectively, at 0 and 30 GPa pressure to highlight the anisotropic character of KGeF_3_ and RbGeF_3_. The isotropy of is represented by the spherical 3D plots, whereas anisotropy is revealed by non-spherical plots^7^. The elastic anisotropy of studied perovskites is appeared in all directions, as indicated by the non-spherical 3D contour plots. The deviation of spherical plots is more extreme at 30 GPa pressure than that exhibited at 0 GPa pressure, manifesting that the applied pressure may promote the anisotropy of AGeF_3_ (A = K, Rb).

## Conclusions

The physical characteristics of lead-free halide perovskites AGeF_3_ (A = K, Rb) under hydrostatic pressure are investigated using DFT. The lattice constant and cell volume reveal similarities with available studies, but decrease with the application of pressure. With increased pressure, the band gap narrows considerably, resulting in improving optical functions and make the compounds suitable for solar cell applications. The ionic/covalent bonds in the compounds also become stronger under pressure. Both compounds exhibit ductile nature at ambient pressure, as determined by their Cauchy pressure, Poisson’s ratio, and Pugh’s ratio. The compounds become more ductile because of pressure effect. The anisotropic nature of both compounds demonstrates similar nature as ductility. At last, it can be expected that this literature will shed fresh light on the improvement of perovskite solar cells and their prospective applications.

## Computational method

The present computations are done by Cambridge Serial Total Energy Package (CASTEP) grounded on density functional theory (DFT)^66^. The orbital shape approximations are not taken into account in the CASTEP code^67^. Though the compounds KGeF_3_ and RbGeF_3_ are yet to synthesize their crystal structure is constructed by taking the reported crystallographic data determined by the theoretical investigation^20^. Houari et al.^20^ predicted that both compounds may have cubic perovskite-type structure with the space group *Pm-*3*m* (#221) as well as the lattice constant is 4.46 Å and 4.49 Å for KGeF_3_ and RbGeF_3_, respectively. The generalized gradient approximation (GGA) combined with Perdew–Berke–Emzerhof (PBE) functional is chosen to perform the exchange–correlation effect^68^. To evaluate the electron–ion interaction, the Vanderbilt-type ultrasoft pseudopotential is selected^69^. The cut off energy is 900 eV following a *k*-point grid of 12 × 12 × 12. To sample the Brillouin zone, the Monkhorst–Pack scheme^70^ is considered. The convergence tolerance factors are set as 5 × 10^–6^ eV/atom for total energy, 5 × 10^–4^ Å for maximum displacement, 0.01 eV/Å for maximum force, and 0.02 GPa for maximum stress. The Broyden–Fletcher–Goldferb–Shanno (BFGS) algorithm^71^ is employed to optimize the crystal structure. The relatively similar approximations were also employed to optimize the crystal structure of experimentally synthesized Pb-based and Pb-free cubic halide perovskites^39,72–74^. In this study, the hydrostatic pressure up to 30 GPa with an interval of 10 GPa is applied during structural optimization. The optimized crystal structure is constructed by VESTA software^75^. The electronic and optical properties are calculated by using the same parameters that are utilized in structural optimization. The elastic constants and elastic moduli are determined by “stress–strain” method^76^ embodied in the CASTEP code. The ELATE program^77^ is used to create the three-dimensional (3D) anisotropic contour plots of Young’s modulus, shear modulus, and Poisson’s ratio.

## Supplementary Information

Supplementary Figures.

## Data Availability

The datasets generated and/or analyzed in this study are available from the corresponding author upon reasonable request.
