# Supplementary material for: Tuning band gap and enhancing optical functions of AGeF3 (A = K, Rb) under pressure for improved optoelectronic applications
Source: Sci Rep. 2022 May 23;12:8663. doi: 10.1038/s41598-022-12713-4 (PMC9126918; doi:10.1038/s41598-022-12713-4)
Supplement: Supplementary file 1 — Supplementary Figures. [file 41598_2022_12713_MOESM1_ESM.docx]

**Supporting information**

**Tuning band gap and enhancing optical functions of AGeF_3_ (A = K, Rb) under pressure for improved optoelectronic applications**

**Md. Safin Alam^a^, Md Saiduzzaman^a*^, Arpon Biswas^a^, Tanjun Ahmed^a^, Aldina Sultana^a^, Khandaker Monower Hossain^b*^**

*^a^Department of Materials Science and Engineering, Khulna University of Engineering & Technology (KUET), Khulna-9203, Bangladesh*

*^b^Department of Materials Science and Engineering, University of Rajshahi, Rajshahi-6205, Bangladesh*

Corresponding author

E-mail: [monower37@gmail.com](mailto:monower37@gmail.com) (K. M. Hossain), [msaiduzzaman@mse.kuet.ac.bd](mailto:msaiduzzaman@mse.kuet.ac.bd) (M. Saiduzzaman)


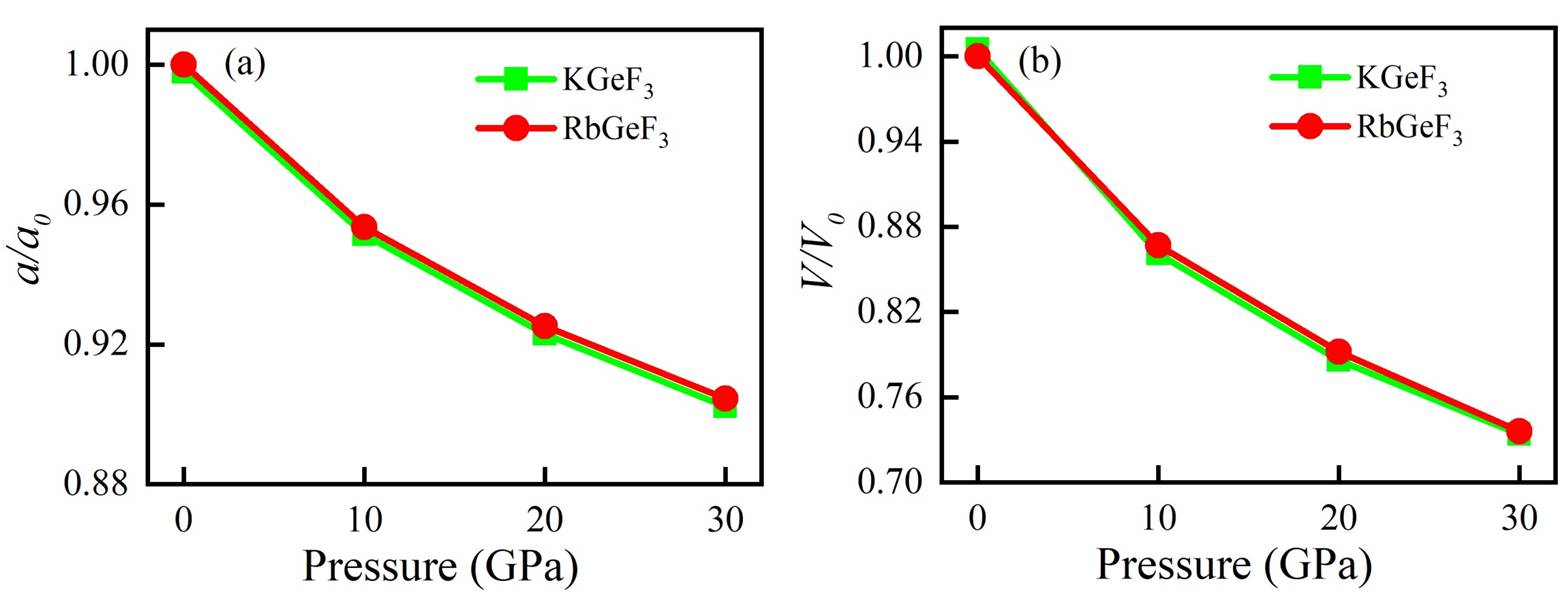


**Fig. S1.** The change of **(a)** lattice constant and **(b)** unit cell volume of AGeF_3_ (A = K, Rb) under pressure.


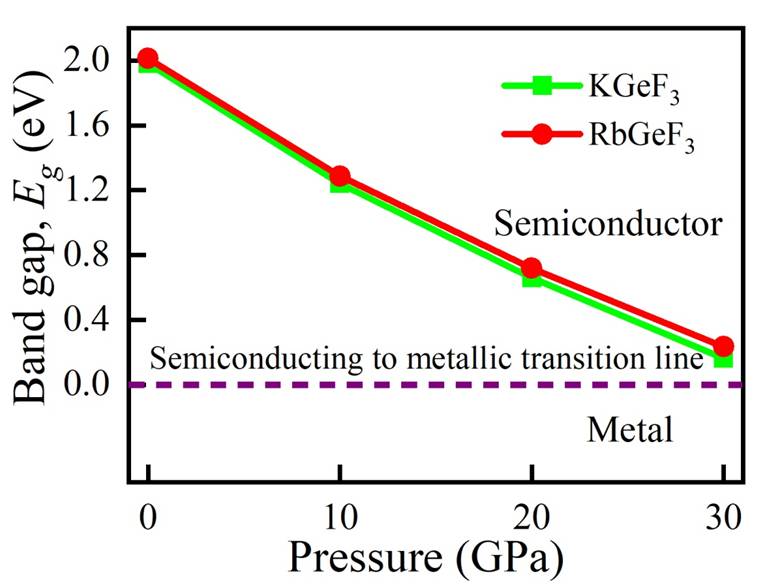


**Fig. S2.** Band gap narrowing of AGeF_3_ (A = K, Rb) under applied pressure.


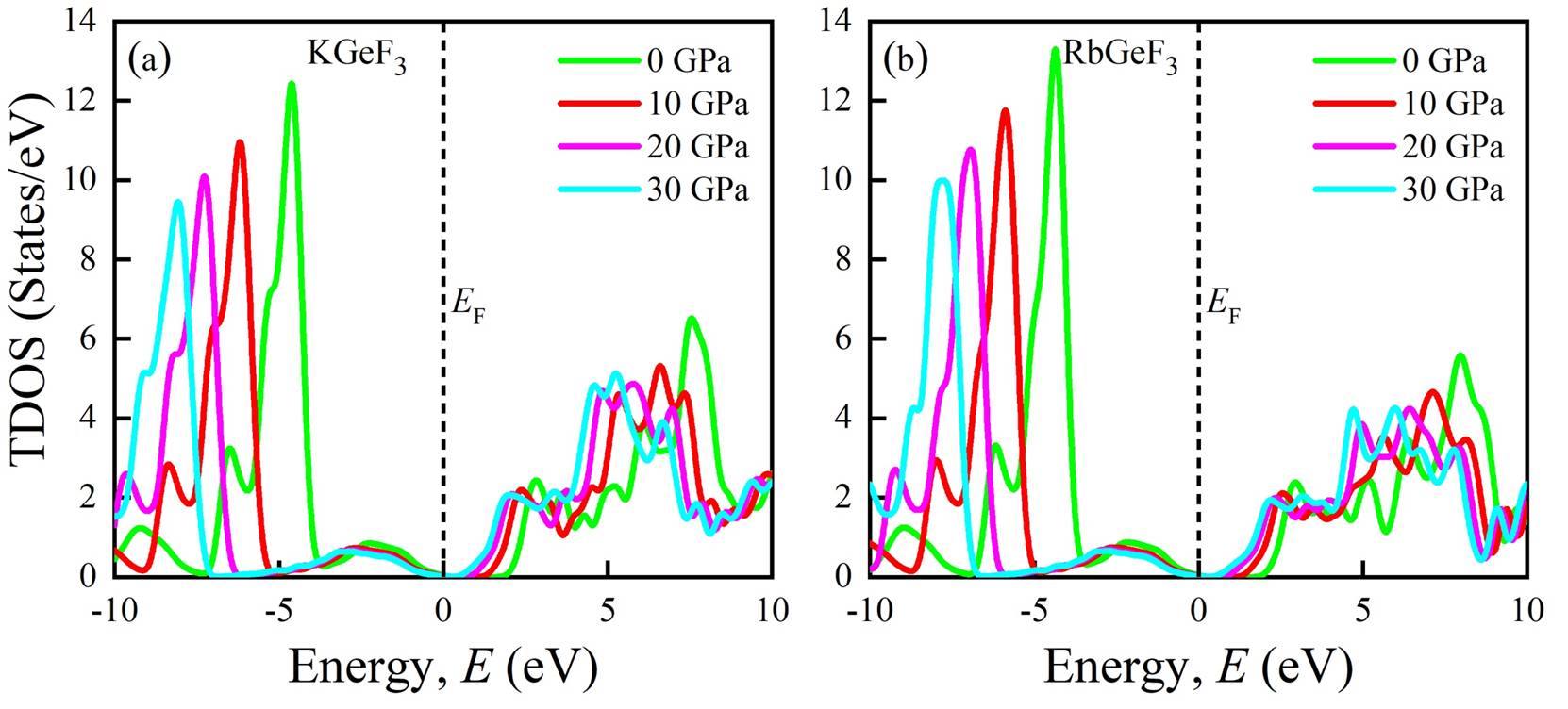


**Fig. S3.** Total density of states of **(a)** KGeF_3_ and **(b)** RbGeF_3_ under applied pressure.


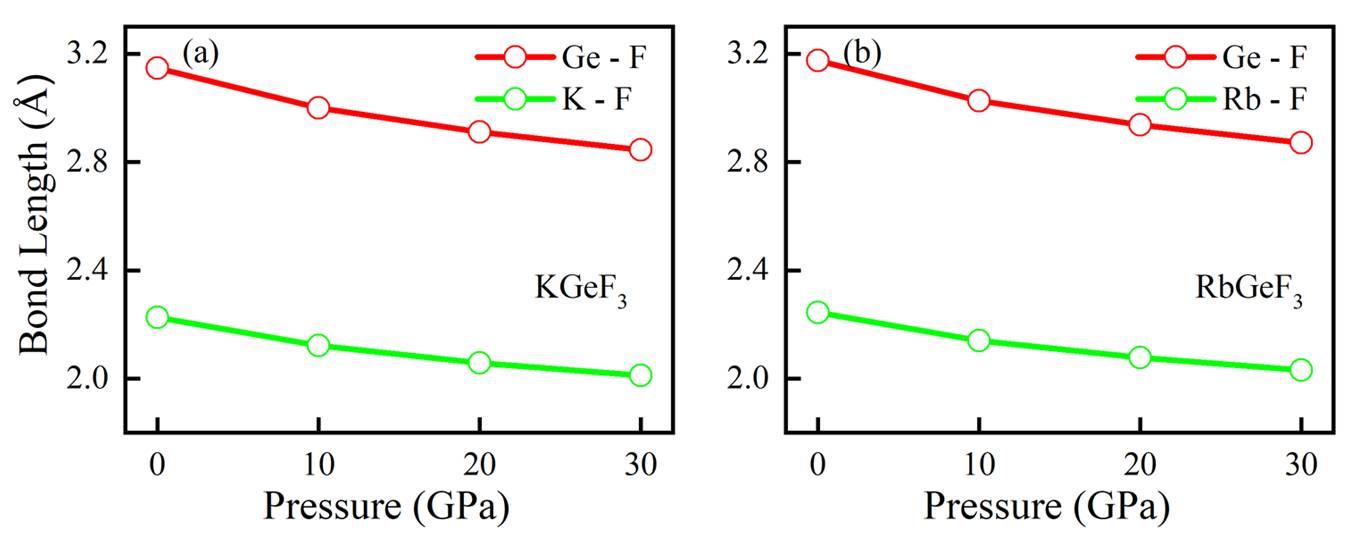


**Fig. S4.** Influence of pressure on the bond length of **(a)** KGeF_3_ and **(b)** RbGeF_3_.


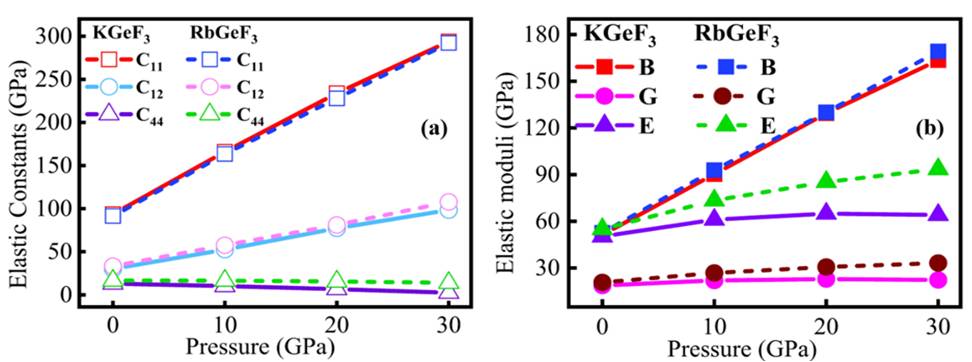


**Fig. S5.** Pressure dependence of **(a)** elastic constants (*C*_11_, *C*_12_, and *C*_44_) and **(b)** elastic moduli (*B*, *G*, and *E*) for AGeF_3_ (A = K, Rb).


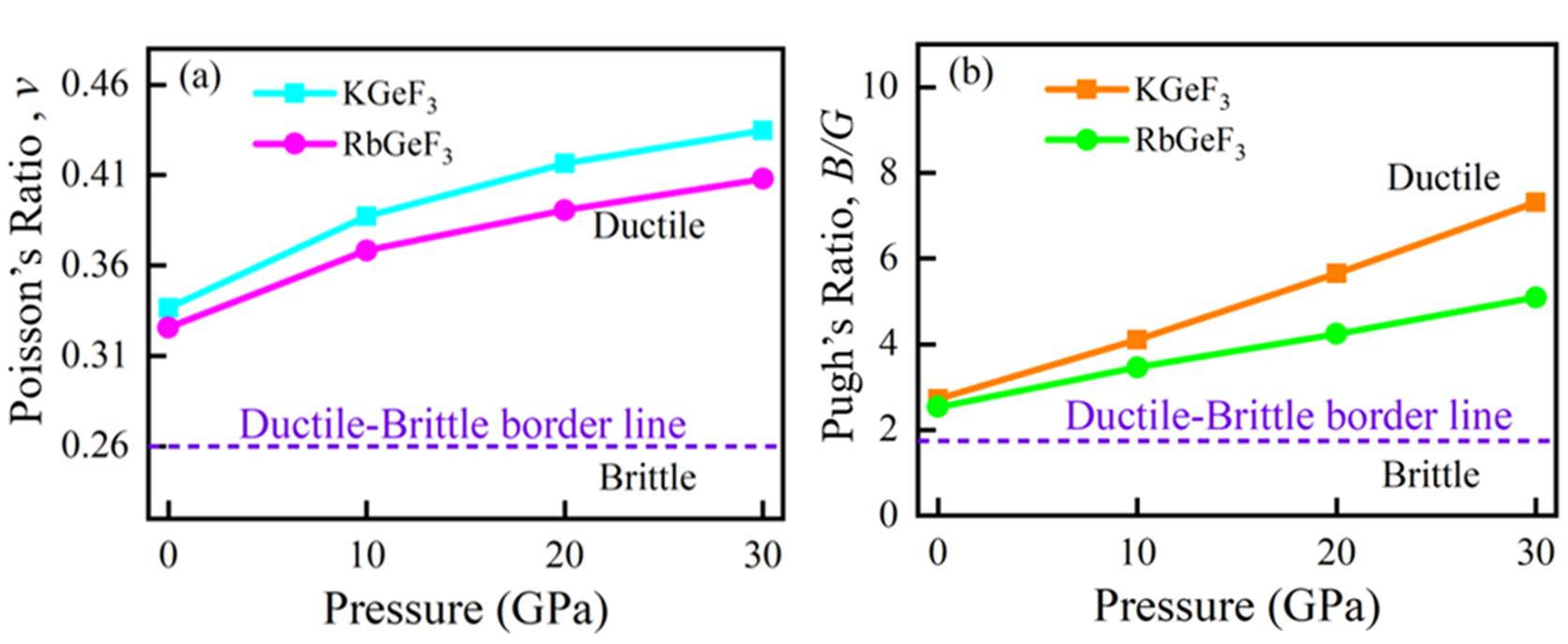


**Fig. S6.** Variation of **(a)** Poisson’s ratio and **(b)** Pugh’s Ratio of AGeF_3_ (A = K, Rb) under pressure.
